# Functional Analysis of Hif1 Histone Chaperone in *Saccharomyces cerevisiae*

**DOI:** 10.1534/g3.118.200229

**Published:** 2018-04-16

**Authors:** Nora S. Dannah, Syed Nabeel-Shah, Christoph F. Kurat, Sarah A. Sabatinos, Jeffrey Fillingham

**Affiliations:** * Department of Chemistry and Biology, Ryerson University, 350 Victoria St., Toronto, M5B, 2K3 Canada; † Molecular Biology Division, Biomedical Center, Faculty of Medicine, LMU Munich, 82152 Planegg-Martinsried, Germany

**Keywords:** H3/H4 chaperone, NASP, chromatin, Asf1, SHNi TPR, Hif1p, Hat1

## Abstract

The Hif1 protein in the yeast *Saccharomyces cerevisie* is an evolutionarily conserved H3/H4-specific chaperone and a subunit of the nuclear Hat1 complex that catalyzes the acetylation of newly synthesized histone H4. Hif1, as well as its human homolog NASP, has been implicated in an array of chromatin-related processes including histone H3/H4 transport, chromatin assembly and DNA repair. In this study, we elucidate the functional aspects of Hif1. Initially we establish the wide distribution of Hif1 homologs with an evolutionarily conserved pattern of four tetratricopeptide repeats (TPR) motifs throughout the major fungal lineages and beyond. Subsequently, through targeted mutational analysis, we demonstrate that the acidic region that interrupts the TPR2 is essential for Hif1 physical interactions with the Hat1/Hat2-complex, Asf1, and with histones H3/H4. Furthermore, we provide evidence for the involvement of Hif1 in regulation of histone metabolism by showing that cells lacking *HIF1* are both sensitive to histone H3 over expression, as well as synthetic lethal with a deletion of histone mRNA regulator *LSM1*. We also show that a basic patch present at the extreme C-terminus of Hif1 is essential for its proper nuclear localization. Finally, we describe a physical interaction with a transcriptional regulatory protein Spt2, possibly linking Hif1 and the Hat1 complex to transcription-associated chromatin reassembly. Taken together, our results provide novel mechanistic insights into Hif1 functions and establish it as an important protein in chromatin-associated processes.

Histone chaperone proteins have key roles in eukaryotic chromatin dynamics (De Koning *et al.* 2007; Keck and Pemberton 2012; Burgess and Zhang 2013). Several classes of these histone chaperones are grouped according to their substrate binding specificities as well as sequence and structural homology (De Koning *et al.* 2007; Eitoku *et al.* 2008). The NASP (**n**uclear **a**utoantigenic **s**perm **p**rotein) family of H3/H4 histone chaperones is characterized by a conserved motif architecture of four tetratricopeptide repeats (TPR), where the second TPR is typically interrupted by a stretch of acidic residues (Dunleavy *et al.* 2007; Nabeel-Shah *et al.* 2014).

In mammals, expression of *NASP* is essential for viability (Richardson *et al.* 2006) and its transcript is found predominantly as one of two alternatively spliced isoforms (Richardson *et al.* 2000). The longer version is expressed in the testis and embryonic tissues and is known as testicular NASP (tNASP). The smaller form, somatic NASP (sNASP), lacks 339 amino acids and is ubiquitously expressed in dividing cells (Richardson *et al.* 2000). The human sNASP protein is a component of the cytosolic histone acetyltransferase-1 (HAT1) complex, which acetylates newly synthesized histone H4 at specific lysine (K) residues 5 and 12 before deposition into chromatin (Allis *et al.* 1985; Parthun *et al.* 1996; Campos *et al.* 2010). Acetylated H4 substrates join histone H3 and are transferred to H3/H4 chaperone Asf1 (anti-silencing factor 1) via a poorly understood physical interaction with the Hat1-complex including sNASP (Campos *et al.* 2010; Alvarez *et al.* 2011). An Asf1 and NASP physical interaction is reportedly conserved in several eukaryotes including ciliate *Tetrahymena thermophila*, yeast *Saccharomyces cerevisiae* and humans (Fillingham *et al.* 2008; Campos *et al.* 2010; Garg *et al.* 2013).

Hat1-interacting factor-1 (Hif1) is the NASP homolog in *S. cerevisiae*. Hif1 was initially identified as a nuclear protein that physically interacts with the Hat1/Hat2-complex, forming a heterotrimeric complex that was subsequently named the ***nu***clear type-***B*** HAT complex for the acetylation of free histone H***4*** (NuB4) (Poveda *et al.* 2004; Ai and Parthun 2004). Within this heterotrimeric complex, Hat1 is the enzymatic subunit responsible for mediating histone acetyltransferase activity. The yeast Hat2 is a homolog of human RbAP46/48 proteins. The three subunits of the NuB4 complex (Hat1, Hat2 and Hif1) are found at roughly stoichiometric levels and Hif1-Hat1 interaction is bridged by Hat2 (Poveda *et al.* 2004; Ai and Parthun 2004). Although early studies suggested that Hif1 is exclusively a nuclear protein (Poveda *et al.* 2004; Ai and Parthun 2004), recent evidence indicates that it might also function in cytoplasm (Blackwell *et al.* 2007; Campos *et al.* 2010). Deletion of *HIF1* results in defective telomere silencing and DNA double strand break (DSB) repair (Ai and Parthun 2004; Ge *et al.* 2011). These defects significantly resemble those that are observed in *hat1* null mutants suggesting a functionally linked role of Hat1/Hif1 in telomeric silencing and DNA double strand break repair (Ai and Parthun 2004; Ge *et al.* 2011). A recent report suggests that Hif1 functions in chromatin assembly via an interaction with specific RNA species (Knapp *et al.* 2014), and exists in protein complexes independently of other Hat1-complex components (Ge *et al.* 2011).

The *Schizosaccharomyces pombe* NASP homolog is known as *Silencing in the middle of the centromere protein* 3 (Sim3) and is a centromere-specific histone chaperone (Dunleavy *et al.* 2007) that is not a component of the Hat1-complex (Tong *et al.* 2012; Kim *et al.* 2014). Sim3 has some functional overlap with Asf1 as a histone H3/H4 chaperone (Tanae *et al.* 2012). These reported differences in Hif1 functions within closely related lineages such as *S. cerevisiae* and *S. pombe* suggest that NASP-family proteins might have experienced functional diversification over the course of evolution, resulting in the acquisition of species or lineage-specific functions.

NASP-family proteins are reported to be functionally important in regulating histone metabolism. Human NASP regulates soluble H3/H4 reservoirs through chaperone-mediated autophagy (Cook *et al.* 2011). In *Xenopus laevis* oocytes NASP homolog N1/N2 functions to buffer soluble histones H3/H4 which are required for DNA replication in the early embryo (Dilworth *et al.* 1987). Similarly, Sim3 functions in the general maintenance of chromatin via its H3/H4 chaperone activities (Tanae *et al.* 2012). A recent report demonstrates an *Arabidopsis* NASP homolog to function as histone H3/H4-specific chaperone (Maksimov *et al.* 2016). Taken together, these studies functionally link NASP-family proteins with chromatin maintenance through dynamic histone regulation. Intriguingly, Hif1 has not been well studied in this capacity thus far, and it´s possible role (s) in regulating histone metabolism is poorly understood.

Recently, we reported a detailed molecular evolutionary analysis of NASP family proteins and established that the acidic residues found within TPR2 are under strong selective pressure and are likely critical for proper function (Nabeel-Shah *et al.* 2014). Human sNASP displays binding specificity for histones via distinct TPR motifs (Wang *et al.* 2012). Recently, Liu and colleagues reported a crystal structure of partial Hif1 protein, and in accordance with our results (Nabeel-Shah *et al.* 2014), they demonstrated that the TPR2 acidic region is essential for Hif1 histone binding (Liu *et al.* 2014). The specific region of Hif1 responsible for its interaction with the Hat1-complex is unknown.

Here we report a functional and molecular evolutionary analysis of Hif1. In order to examine the role of different TPR motifs, we generated several N-terminal MYC tagged Hif1 truncation mutants. Our results show that the acidic patch within TPR2 is essential for Hif1 interactions with the Hat1/Hat2 complex, Asf1 and histones H3/H4. We show that a basic patch at the extreme C-terminus of Hif1 is indispensable for its proper nuclear localization. To understand Hif1 role(s) in histone homeostasis, we carried out a histone over expression assay using various Hif1 truncation mutants. We report that *HIF1* contributes to histone homeostasis, and that a null mutant exhibits growth defects with an increased histone gene dosage. We also found that deletion of *HIF1* results in synthetic lethality when combined with a null mutation of histone mRNA regulator *LSM1*. These results highlight a role of Hif1 in histone metabolism and suggest that, similar to human sNASP, Hif1 might function as a buffer for soluble histones. Finally, to begin to decipher possible functions of Hif1 in transcription regulation, we demonstrate a physical interaction of NuB4 complex with the transcriptional regulatory protein Spt2. We discuss the implications of these results and provide a framework for various chromatin related functions of Hif1.

## Materials and methods

### Phyogenetic analysis

We utilized our previously published raw data (Nabeel-Shah *et al.* 2014) to compare the distribution of NASP homologs among fungi. Domain analysis of the identified sequences was carried out using Pfam database (http://pfam.sanger.ac.uk/) in order to gauge the presence of the NASP signature motif (SHNi-TPR, PF10516). Multiple sequence alignments were built using MUSCLE under default parameters (Edgar 2004). TPR motifs in identified orthologs were predicted using Hif1 structural data (Liu *et al.* 2014) and human NASP functional data (Wang *et al.* 2012). Phylogenetic analysis was conducted using maximum likelihood (ML, MEGA5 (Tamura *et al.* 2011)) and Bayesian methods (Mr. Bayes v3.2.0 (Ronquist *et al.* 2012). We used conserved sequences of TPR motifs, and the model rtREV+G+F which best fits the sequence data were selected using model selection option as implemented in MEGA5. The robustness of the resulting ML trees was assessed by a bootstrap method with 200 replicas. Posterior probabilities from two independent runs of one million generations (0.25 burn-in frequency) were used as an indicator of tree reliability in Bayesian analysis.

The degree of structural conservation was calculated using the program ConSurf (Ashkenazy *et al.* 2016) based on Hif1 structural data (PDB ID: 4NQ0) (Liu *et al.* 2014) and multiple sequence alignments of amino acids of the identified fungal homologs.

### Gene network analysis

Yeast genetic and protein interactions data (Krogan *et al.* 2006; Collins *et al.* 2007a, 2007b; Fiedler *et al.* 2009) were downloaded from the BIOGRID database (Stark *et al.* 2006 and references therein). A gene/protein interaction network was built using GeneMania algorithm (Warde-Farley *et al.* 2010) as implemented in cytoscape web application. Networks were constructed and visualized using cytoscape version 3.1.1 (Cline *et al.* 2007).

### Strains used in the study

*S. cerevisiae* strains used in this study were generated through standard molecular genetic procedures and are listed in Supplementary Table S1. Primer sequences used for overlapping polymerase chain reactions are listed in Supplementary Table S2. Tetrad analysis was carried out using standard methods. A total of 9 tetrads were dissected for each cross as indicated in text.

### Extraction of proteins and Western blots

Whole cell extracts were prepared using trichloroacetic acid (TCA) as described previously (Kao and Osley 2003). Proteins were separated using 10% SDS-PAGE, transferred onto nitrocellulose paper, and immunoblotted with antibodies mouse monoclonal IgG α-MYC (Santa Cruz Biotechnology) primary antibody, or rabbit monoclonal α-TAP (Thermo scientific) primary antibody at 1:7500 dilutions. Equivalent loading in each lane was assessed by Ponceau S.

### Immunoprecipitations (IPs)

IPs were performed as previously described (Kobor *et al.* 2004). Histone IPs were carried out essentially as described above with the following modifications: 2 µg of anti-acetyl H4 antibody (Abcam: ab46983) was conjugated with 25 µl of protein G Dynabeads (Thermo Fisher Scientific: 10003D) for one hour at room temperature. The antibody-conjugated beads were then incubated with the cell lyses for overnight at 4°.

### Indirect immunofluorescence

Indirect immunofluorescence was performed using a modified method of (Poveda *et al.* 2004). Briefly, cells grown to mid log phase (∼2 × 10^7^) were fixed in 4% formaldehyde for 60 min with gentle agitation at room temperature. Cells were washed in 1x PBS twice and resuspended in 500 μL of Spheroplasting Buffer (2% Glucose, 1x Amino Acids, 1x Sorbitol, 20 mM Tris-HCl pH 7.5 and 1x YNB), and kept at 4° overnight. Next morning, 10 mg/ml zymolyase 100T and 1.42 M beta-mercaptoethanol were added to 200μL of cells in spheroplasting buffer, and incubated at 30° for 90 min. Cells were then washed twice with 1mL of 1xPBS +0.05% Tween20 and suspended in 1xPBS +0.05% Tween20. The cell suspension was added to poly-D-lysine coated cover slips and dried for 20 min followed by three washes with 1x PBS. 1xPBS +1 mg/mL BSA blocking buffer was added for 30 min, and were then washed three times with 1xPBS. Primary antibody (anti-Myc) was added in a 1:250-dilution in 1xPBS+1mg/mL BSA at room temperature for 1 hr in a humid chamber. After three washes with 1xPBS, fluorescent secondary antibodies (anti-mouse, Alexa488) in a 1:1000 dilution in 1xPBS+1mg/mL BSA were incubated in the dark for 45 min. Subsequently, the cover slips were washed three times with 1xPBS, and incubated with 1:5.000 diluted DAPI in 1xPBS for 10 min. Coverslips were then washed three times with 1xPBS prior to adhering the cover slips onto a clean slide with Fluorescent Mounting Media (Dako). Microscopy was performed using Olympus Spinning Disk Confocal Microscopy at the Sick Children Hospital Bio-Imaging Facility.

### Spot assays for DNA-damage sensitivity and histone Over-expression

Yeast strains were inoculated in 5ml YPD medium and were grown overnight at 30° within shaking. The optical density of each culture was measured and adjusted to OD600nm = 0.2. Cells were then grown to OD600nm = 0.5, and then 1/4 serial dilutions of each cell culture were spotted onto plates containing 50 mM or 100 mM hydroxyurea. Plates were incubated at 30° for 3 days.

For histone over-expression assay, strains were transformed with tagged histone H3 under the control of a galactose-inducible *GAL1-10* promoter or the empty vector control (pYES2). Cells were grown overnight in 5 ml minimal medium (YNB) either minus uracil or minus uracil/minus leucine and containing 2% raffinose. Expression of H3 was induced by addition of 2% galactose for about four hours, and cultures were then grown to approximately OD600nm = 0.8. Sixfold serial dilutions of each strain were performed and cell culture were spotted onto plates containing minimal media either lacking uracil or lacking both uracil and leucine, with either glucose (H3 = OFF) or galactose (H3 = ON) as carbon source. Plates were incubated at 30° for 3 days.

### Data availability

All yeast strains used in this study are available upon request. File S1 contains detailed descriptions of all the supplemental data, including multiple sequence alignments of TPRs 1-4 used to generate the phylogenetic tree. Supplemental material available at Figshare: https://doi.org/10.25387/g3.5969260.

## Results

### Hif1 domain organization is conserved among fungi

**Figure 1:**
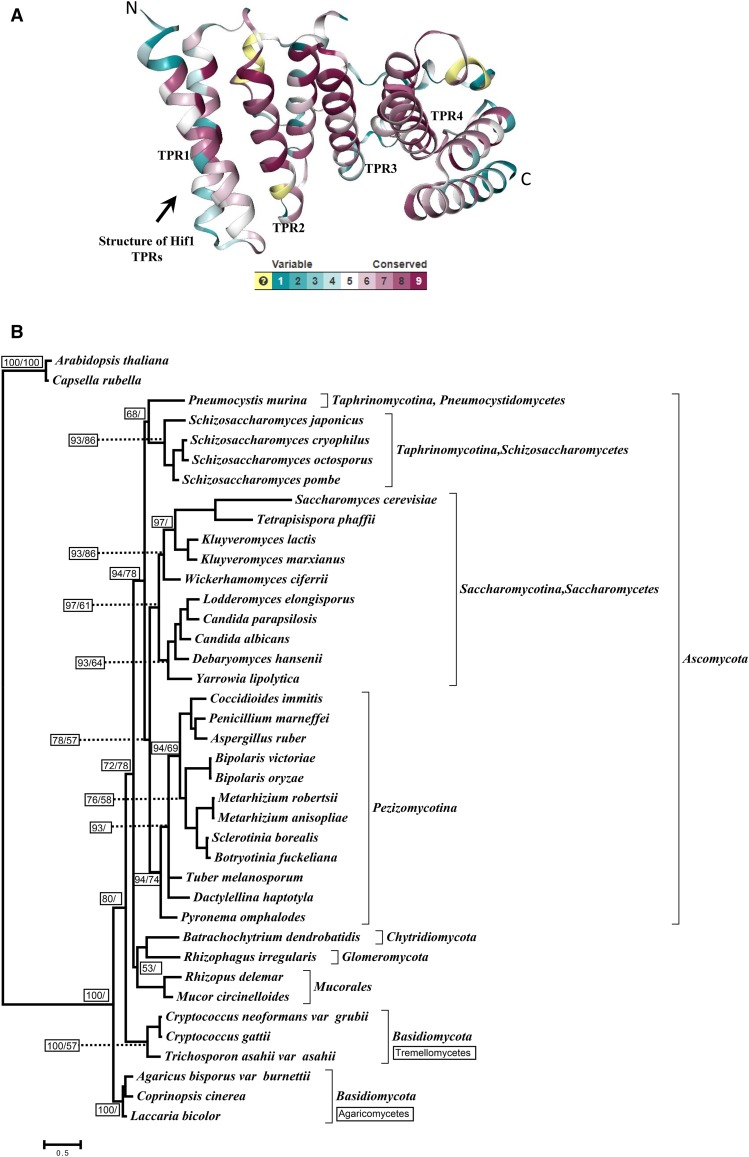
Structural conservation of Hif1 and Phyogenetic analysis. A: Cartoon representation of Hif1 structure (PDB ID: 4NQ0) indicating the degree of conservation as calculated by the program ConSurf based on sequence alignment of fungal homologs. Conservation key is provided with 9 being the most conserved and 1 being the most variable. Note: The light yellow color represents the regions where conservation was not deteremined. B: The phylogenetic tree was reconstructed using TPR 1–4 amino acid sequences and tree topology and branch lengths are based on Bayesian inferences. The average standard deviation of split frequencies from two runs was 0.006. Confidence values are provided in small boxes. The Posterior probability values are indicated on the left within small boxes whereas bootstrap values (based on 200 replicates) for the ML tree are shown on the right (reported only when ≥ 50%). Different fungal groups are indicated in the right margin. The scale bar shows the number of substitutions per site. The tree was rooted using *Capsella rubella* and *Arabidopsis thaliana* as out groups.

We have previously reported that NASP-family proteins are widely distributed throughout eukaryotes (Nabeel-Shah *et al.* 2014). To provide a more comprehensive view of NASP distribution in fungi we used our published raw data (Nabeel-Shah *et al.* 2014), finding NASP orthologs in each major fungal group including *Chytridiomycota*, *Glomeromycota*, *Ascomycota*, *Basidiomycota*, and *Mucorales* (see Supplementary Table S3 for accession numbers). Using the reported structural data for *S. cerevisiae* Hif1 (Liu *et al.* 2014), we predicted the existence of four TPR motifs in the identified fungal homologs. We estimated the structural conservation by mapping the amino acid sequences of the identified homologs on the reported structure of *S. cerevisiae* Hif1 (Liu *et al.* 2014). We observed that functionally important TPR domains are conserved among newly identified homologs (Figure 1A; Figure S1A). In agreement with our previous study (Nabeel-Shah *et al.* 2014), we found that N- to C-terminal organization of TPR1 to 4 is maintained, and that an acidic patch frequently interrupts the TPR2 (Figure S1A-E). Interestingly, we also observed a highly conserved basic patch at the extreme C-termini of all the identified fungal orthologs, similar to a consensus nuclear localization signal (NLS) (Figure S2). This observation suggests that fungal NASP orthologs might have similar sub-cellular localization patterns.

Inferring a protein’s evolutionary history can provide useful insights into functional diversity between different lineages. To extend our previous study (Nabeel-Shah *et al.* 2014), we reconstructed a protein phylogeny using TPRs1-4 sequences from representative fungal lineages. We compared maximum liklihood and Bayesian methods to ensure that our results are not biased by methodological choices. Clustering in the resulting phylogenetic tree (Figure 1B) is mostly in agreement with the known classification system of the major fungal groups (Adl *et al.* 2012) with the notable exception of some *Basidiomycota* lineages (Figure 1B). In particular, we observed that *Agaricomycetes* and *Tremellomycetes* (both *Basidiomycota*) form two distinct groups on the phylogenetic tree with the former appearing relatively more divergent. This observation suggests the existence of possible functional diversity that might be found within closely related *Basidiomycota* lineages. The overall phyletic patterns suggest that the identified sequences in fact share a common ancestry and might have been subject to lineage-specific constraints (Figure 1B) (Figure S2).

NASP family proteins commonly feature an overall net negative charge due to a large number of acidic residues within TPR2 (Nabeel-Shah *et al.* 2014). Remarkably, the number of acidic residues interrupting TPR2 varies greatly within different groups of NASP orthologs. We observed that *Saccharomycetes* consistently have larger acidic stretches compared to other lineages such as *Schizosaccharomycetes* (see Figure S1F). For example, *S. cerevisiae* and *Kluyveromyces lactis* (both *Saccharomycetes*) carry 42 and 53 acidic residues within TPR2 interruption, respectively, whereas *S. pombe* and *S. cryophilus* (both *Schizosaccharomycetes*) have 20 and 23 acidic residues, respectively. Several other features were divergent among different lineages. For example, small insertions in TPR4 are variable in several lineages (see Figure S1G). Notably, within *Ascomycota* lineages the length of TPR4 interruption is highly variable. *Ascomycota* lineages *Schizosaccharomycetes* and *Saccharomycetes* have relatively smaller insertions of approximately 10 or less residues, *e.g.*, *S. pombe* Sim3 carries a 5-residue TPR4 insertion. Other *Ascomycota*, *Euascomycetes* and *Dothideomycetes*, have larger TPR4 insertions (∼30 residues) that include acidic (glutamic and asparatic acid) or serine/threonine amino acids, potentially providing phosphorylation targets. Taken together, the observed differences in the number of TPR2 acidic residues, as well as variable length insertions of TPR4, suggests there may exist functional diversity among fungal Hif1 homologs.

### Hif1 interacts With Hat1/Hat2 via acidic region of TPR2

Several studies have previously established that *S. cerevisiae* Hif1 physically interacts with the Hat1/Hat2-complex presumably via Hat2 (for review see 42). However, the specific region on Hif1 that might be responsible for this interaction has not yet been identified. Our evolutionary analysis (refer to Figures) indicates that Hif1 has a conserved pattern of four TPR motifs, an acidic region which disrupts the TPR2, and a C-terminal putative NLS. These conserved regions provide candidate functional units that might be involved in an interaction with the HAT1-complex. Therefore, to map the Hat1 interaction region in *S. cerevisiae* Hif1 we generated a series of truncation mutants lacking either a specific TPR1-4 motif, or the first alpha helix of TPR2 (before start of the acidic region), or only the acidic region (Figure S3A). We also generated five C-terminal deletion mutants lacking either the NLS, or the last 35 C-terminal residues (including the NLS), or the whole C-terminus until the TPR4, or the C-terminus including TPR4, or the C-terminus until TPR2 (Figure S3A). Wild type and mutant Hif1 proteins were N-terminally tagged with 12×MYC and were transformed in *∆hif1* yeast cells that endogenously expressed C-terminally tagged Hat1 TAP-tag (where TAP is tandem affinity purification). We maintained transformed plasmids (pRB415-12×MYC) using a LEU2 marker on media without leucine. Subsequently, the expression of 12MYC-*HIF1* mutants as well as *HAT1*-TAP was assessed on Western blots using anti-MYC and anti-TAP antibodies, respectively, which showed even expression levels (Figure S3 B, C).

**Figure 2:**
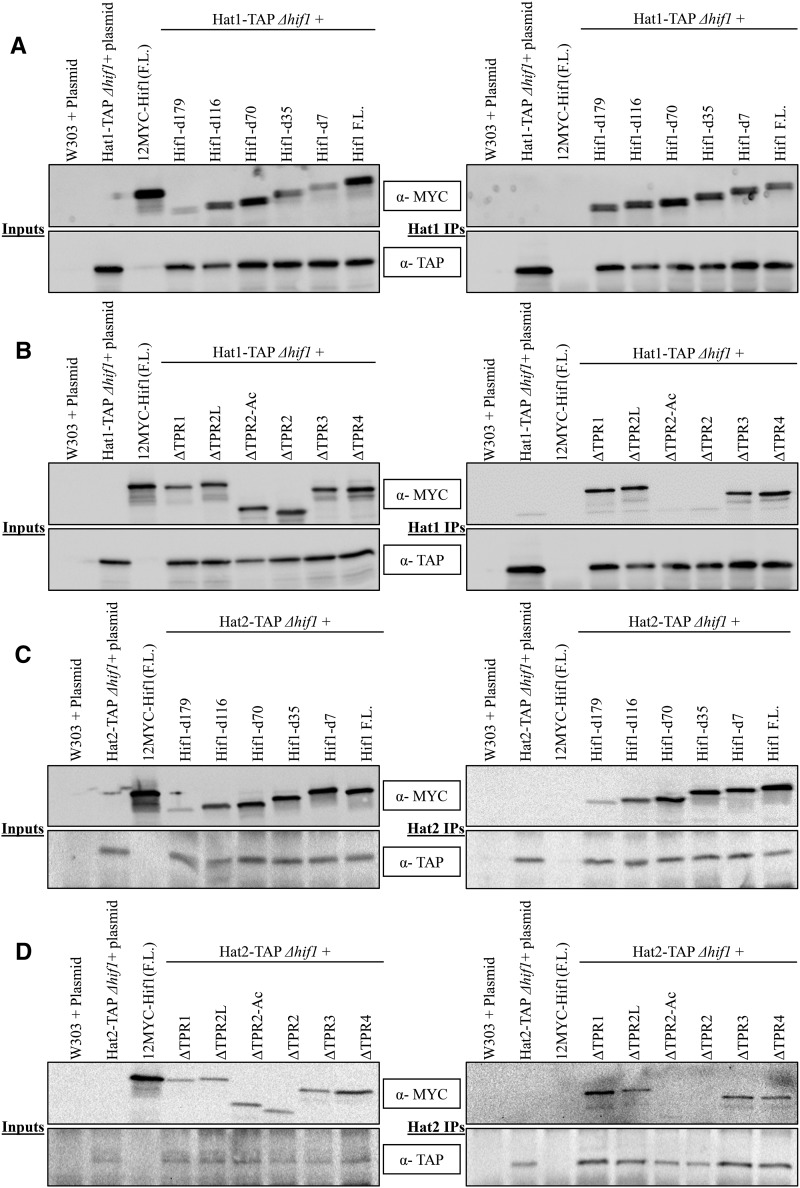
Western blot analysis of Co-IP fractions of Hat1/2-TAP and Hif1 C-terminal (external) and internal deletions constructs. A: *(Left)* Input fractions of Co-IP experiments for various Hif1 C-terminal (external) deletions. *(Right)* Western blot analysis of Co-IP fractions of Hif1 C-terminal mutants to assess their ability to immunoprecipitate with Hat1-TAP B: *(Left)* Input fractions of Co-IP experiments for various Hif1 internal deletion mutants. *(Right)* Co-IP samples of Hif1 internal deletions. C: *(Left)* Input fractions of Co-IP experiments for various Hif1 C-terminal (external) deletions. *(Right)* Western blot analysis of Co-IP fractions of Hif1 C-terminal mutants to assess their ability to immunoprecipitate with Hat2-TAP D: *(Left)* Input fractions of Co-IP experiments for various Hif1 internal deletion mutants. *(Right)* Co-IP samples of Hif1 internal deletions. The red arrows represent the position of HAT2. Note: The size difference of various Hif1 truncated mutants represents various deletions. The top panels were probed with anti-MYC antibody whereas bottom panels were probed with anti-TAP antibody.

In order to identify the Hat1 interacting region of Hif1, we immunoprecipitated Hat1-TAP and probed for 12MYC-Hif1 to confirm that the full length (F.L.) Hif1 and Hat1 proteins interact (Figure 2A). We predicted that 12MYC-Hif1 truncation mutants without the TPR2 acidic region should not co-immunoprecipitate with Hat1-TAP. We began by assessing the ability of C-terminal truncation 12MYC-Hif1 mutants to immunoprecipitate with Hat1-TAP. We observed that none of the five C-terminal deletion mutations affected Hif1-Hat1 interaction, suggesting that the Hif1 C-terminus is not required for this interaction (Figure 2A).

Next, we tested the Hat1-binding abilities of 12MYC-Hif1 mutants carrying internal deletions for a specific TPR motif or only the acidic region. As shown in Figure 2B, internal deletions for either of TPR1, TPR3 and TPR4 did not prevent 12MYC-Hif1 association with the Hat1-TAP. This suggests that none of the three above noted TPR motifs are required for the Hif1-Hat1 interaction. Interestingly, deletion of TPR2 resulted in loss of an interaction (see lane 3 from the right on Figure 2B) indicating that this region is critical for binding with the Hat1-complex. The TPR2 is composed of acidic interruption region that is flanked by two alpha helices (Liu *et al.* 2014). We decided to assess the role of various TPR2 segments in the Hat1 binding. Remarkably, the deletion of TPR2 alpha helix found prior to the start of acidic region did not affect the ability of 12MYC-Hif1 to co-immunoprecipitate with Hat1-TAP (Figure 2B). Conversely, the deletion of the acidic region completely abolished this interaction (see lane 4 from the right on Figure 2B) indicating that Hat1 interaction depends on the acidic region within TPR2.

Hif1 has previously been shown to physically interact with the Hat1/Hat2-complex via Hat2 (Haigney *et al.* 2015). To further characterize the Hif1 interaction with the Hat1/Hat2-complex, we extended our Co-IP analysis and mapped the Hat2 interaction region on Hif1 (Figure 2C, D). As expected, we observed that the deletion of the TPR2 completely abolished Hif1-Hat2 interaction (Figure 2D). Similar to what we observed for Hat1 (see above), the mutant lacking the TPR2 acidic region resulted in the loss of Hif1-Hat2 interaction. In contrast neither the C-terminal deletion mutations nor the mutations carrying internal deletions for TPR1, TPR3 and TPR4 abolished the Hif1-Hat2 interaction indicating that these regions are not required (Figure 2C, D). Taken together these results demonstrate that the acidic region that interrupts TPR2 is essential for Hif1 interaction with the Hat1/Hat2-complex.

### Hif1 interacts With histones H3/H4 via acidic region of TPR2

**Figure 3:**
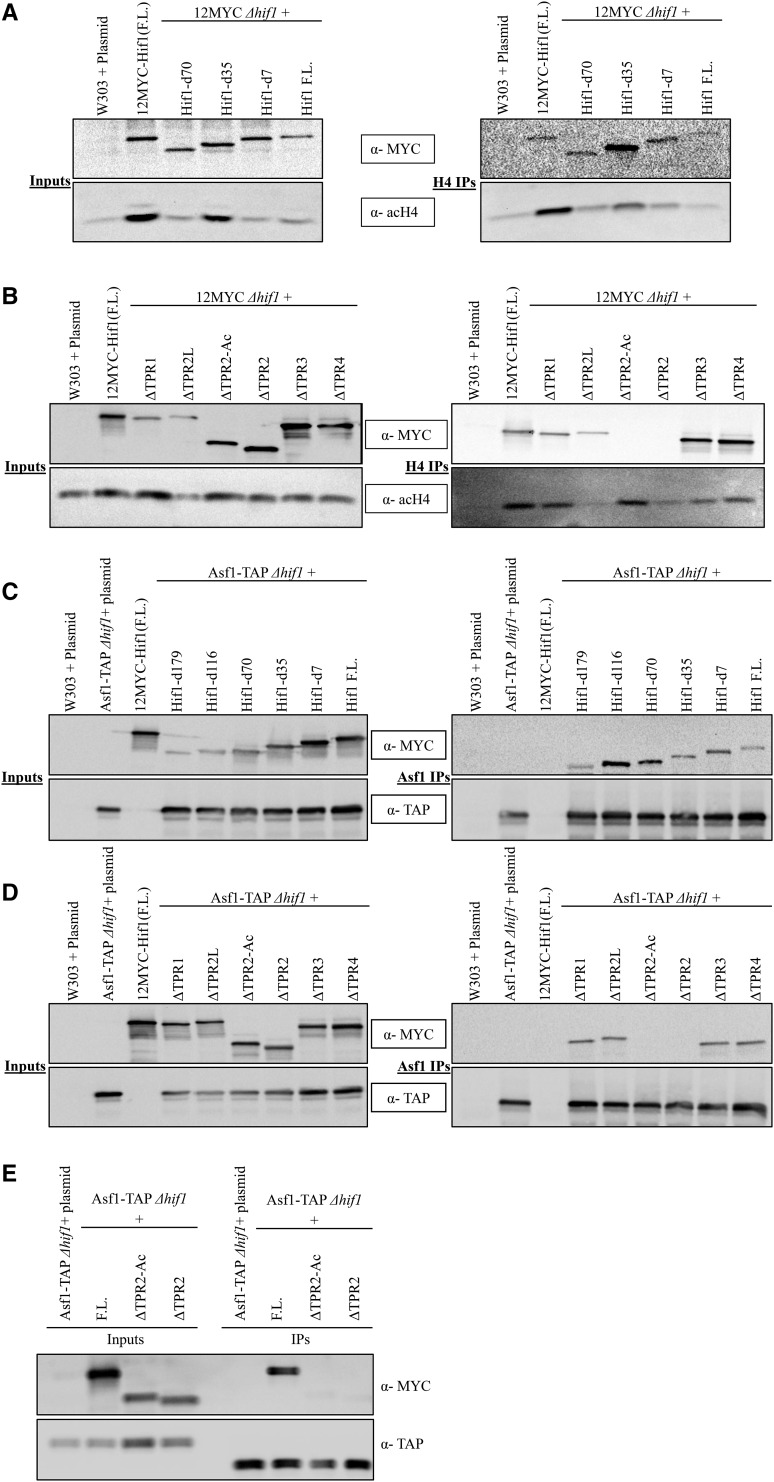
Co-IP analysis of Hif1 truncation mutants and histone H4/ Asf1. A: *(Left)* Input fractions for various Hif1 C-terminal (external) deletions. *(Right)* Western blot of Co-IP fractions of Hif1 C-terminal mutants to monitor their ability to co-immunoprecipitate with acetylated H4. B: *(Left)* Input fractions of Co-IP experiments for Hif1 internal deletion mutants. *(Right)* Co-IP samples of Hif1 internal deletions to examine their ability to come down with acetylated H4. C: *(Left)* Input fractions for various Hif1 C-terminal (external) deletions. *(Right)* Western blot analysis of Co-IP fractions of Hif1 C-terminal mutants to assess their ability to immunoprecipitate with Asf1 D: *(Right)* Input fractions of Co-IP experiments for Hif1 internal deletion mutants. *(Left)* Western blot analysis of Co-IP samples of Hif1 internal deletion mutants to assess their ability to immunoprecipitate with Asf1. E: One step co-IP using TEV protease elution of Asf1-TAP was performed in order to obtain better resolution of Asf1-TAP and the shorter Hif1 internal deletion clones (see methods). The Asf1 size difference between inputs and IPs is due to TEV cleavage. Note: The top panels were probed with anti-MYC antibody whereas middle panel was probed with either anti-acH4 or anti-TAP antibody to detect acetylated H4 or Asf1, respectively.

Hif1 has been shown to bind with histones H3/H4 *in vitro* (Ai and Parthun 2004). Furthermore, as a member of the Hat1-complex, Hif1 participates in the acetylation of newly synthesized histone H4 (Campos *et al.* 2010). To investigate the mechanism through which Hif1 binds histones H3/H4 *in vivo*, we carried out Co-IP experiments using the Hif1 mutants described above (see Figure S3A). As in Figure 2, each of the Hif1 mutants were expressed in cells lacking the wild type *HIF1* (Δ*hif1*), and histones were pulled down using an antibody against acetylated H4 (Figure 3A, B). Consistent with what we observed for the Hat1/Hat2, the Hif1 mutants lacking the TPR2 and/or acidic-interruption region failed to immunoprecipitate with the acetylated histone H4 (Figure 3B). In contrast, neither the C-terminal deletion mutations nor the mutations carrying internal deletions for TPR1, TPR3 and TPR4 abolished the Hif1 association with H4 (Figure 3A, B). These results are consistent with a recent study suggesting that Hif1 TPR2 acidic region is important for H3/H4 binding *in vitro* (Liu *et al.* 2014; Zhang *et al.* 2016).

### Hif1 interacts With Asf1 via acidic region of TPR2

Asf1 is a key generalized H3/H4 chaperone that functions in an array of chromatin-related processes including the transport of newly synthesized H3/H4 and chromatin assembly pathways (English *et al.* 2006; Campos *et al.* 2010; Alvarez *et al.* 2011) . Asf1 and Hif1 interaction has been extensively reported where Asf1 is thought to function downstream of Hif1 in the H3/H4 transport pathway (Campos *et al.* 2010). *In vitro* studies have suggested that the interaction between Hif1 and Asf1 is likely mediated by histones H3/H4 (Haigney *et al.* 2015; Bowman *et al.* 2017). To examine what region of Hif1 is required for an interaction with Asf1 *in vivo*, we employed our described Co-IP strategy. We expressed the Hif1 mutants (see Figure S3A) in *hif1∆* cells expressing C-terminally TAP-tagged Asf1 from its endogenous locus. Our Co-IP experiments indicated the TPR2 as the region responsible for the Hif1-Asf1 interaction (Figure 3C-E). Remarkably, we observed that the Hif1 mutant lacking only the acidic region of TPR2 was defective in its ability to immunoprecipitate with Asf1 (Figure 3C-E). In contrast, the C-terminal deletion mutations and the mutations carrying the internal deletions for TPR1, TPR3 and TPR4 were found not to be required for Hif1-Asf1 interaction (Figure 3C-E). These results establish that the acidic region of TPR2 is functionally important for Hif1-Asf1 interaction. Because the TPR2 acidic region is also essential for H3/H4 binding (see above), these results, taken together, suggest that the *in vivo* Asf1-Hif1 interaction likely mediated by H3/H4 consistent with previous *in vitro* (Haigney *et al.* 2015) and *in vivo* studies (Campos *et al.* 2010).

### The conserved basic patch is essential for Hif1 nuclear localization

**Figure 4:**
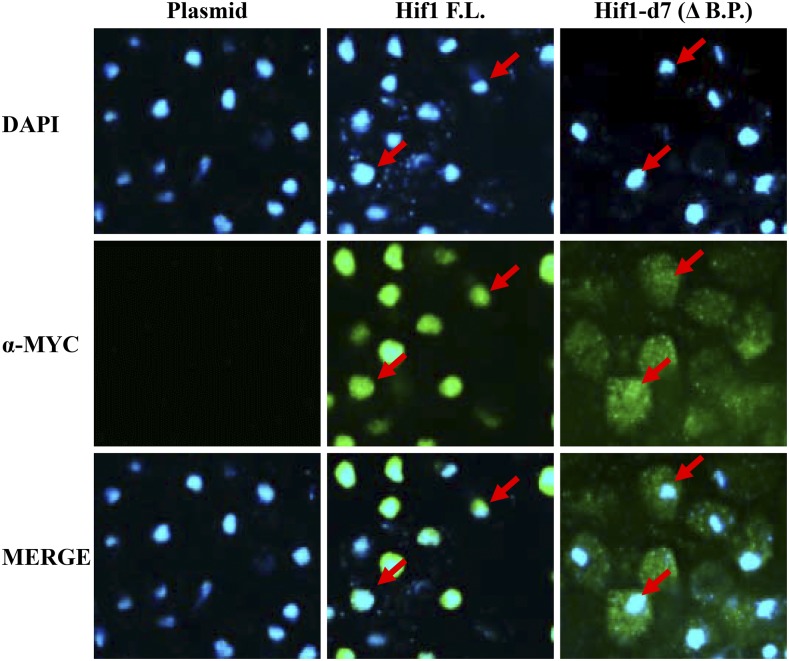
Indirect immunofluorescence analysis of Hif1. Top panel was stained with DAPI to capture the nuclear orientation. Middle panel was probed with anti-MYC antibody to examine the localization of either full length Hif1 (F.L) or truncated mutant lacking basic patch (-B.P). Cells transfected with an empty vector were used as a control. Red arrows point toward the nuclei of representative cells. The bottom panel represents merge of DAPI and anti-MYC staining.

Our molecular evolutionary analysis indicated that Hif1 carries a conserved basic patch at its extreme C-terminus which shares similarity to the consensus NLS (Figure S2). To examine the functionality of the putative NLS we carried out an indirect immunofluorescence (IF) analysis using 12MYC tagged Hif1 cells. In accordance with previous studies (Poveda *et al.* 2004), the full length 12MYC-Hif1 mainly localized to the nucleus (Figure 4). Strikingly, we observed that Hif1 mutant lacking the C-terminal basic patch was mostly located in the cytoplasm indicating that this region is required for the proper nuclear import of the protein (Figure 4). We extended our IF analysis using the Hif1 mutants described above. All Hif1 mutants carrying an internal deletion predominantly localized to the nucleus indicating that eliminating any of these regions does not abolish nuclear localization. Conversely, Hif1 mutants carrying C-terminal external deletions were found to be defective for proper nuclear localization and the signal was observed mostly in the cytoplasm (Figure S4). Together these observations establish that Hif1 C-terminal basic patch is essential for the protein’s nuclear localization consistent with function as an NLS.

### *hif1Δ* mutants are sensitive to histone Over-expression

Excessive soluble histones are known to have deleterious effects on genomic stability (Singh *et al.* 2010; Huddleston 2011). In *S. cerevisiae* excessive histones are immediately degraded via a pathway that includes Rad53 and the Proteasome (Gunjan and Verreault 2003; Singh *et al.* 2009). Previously, Lsm1 has been shown to function in cell cycle based regulation of histone mRNA decay (Herrero and Moreno 2011). Interestingly, NASP-family proteins among chordates have been reported to also be important for regulation of histone metabolism (Dilworth *et al.* 1987; Cook *et al.* 2011). We therefore asked if Hif1 has a role in regulating histone dynamics. We initiated our analysis by assessing the effect of histone over-expression in *hif1*Δ cells. If Hif1 is important for the regulation of histones, we expected *hif1*Δ cells to be sensitive to histone over-expression. We also included *hat1*Δ and *hat2*Δ strains in our analysis since these proteins along with Hif1 form a nuclear NuB4 complex (Parthun 2012). Furthermore, a *lsm1*Δ strain was included because cells lacking *LSM1* have been reported to exhibit hypersensitivity to histone over-expression (Herrero and Moreno 2011). Wild-type, *hif1*Δ, *hat1*Δ, *hat2*Δ and *lsm1*Δ strains were transformed with plasmids encoding histone H3 gene under the control of *GAL1* promoter or with an empty vector. Subsequently, raffinose-containing medium was used to grow the transformants and cell cultures in serial dilutions were plated on glucose (promoter OFF) or galactose (promoter ON)-containing media.

**Figure 5:**
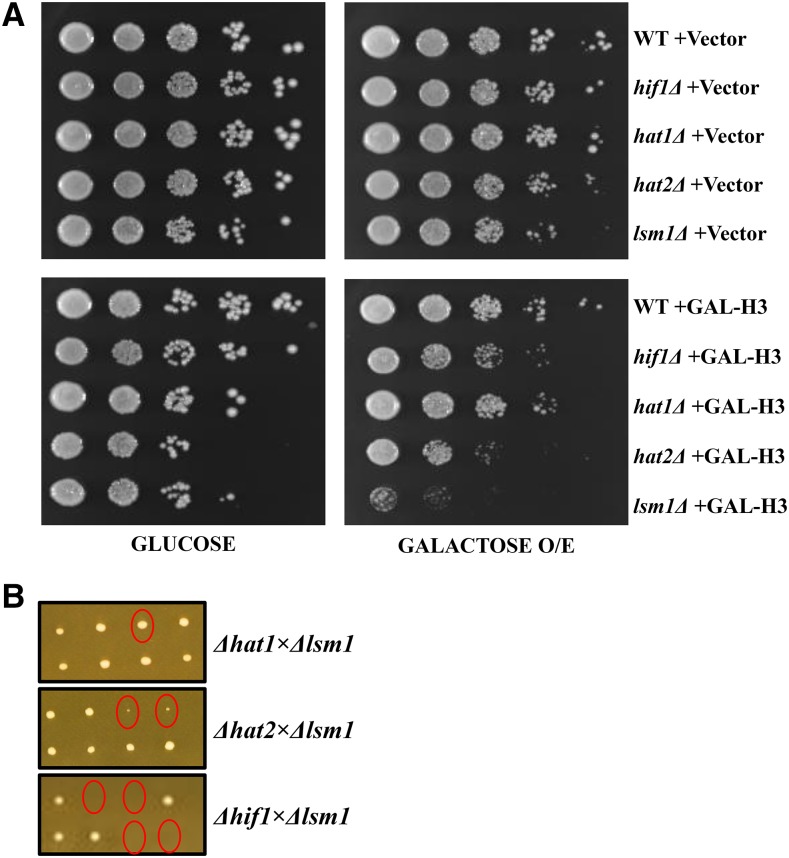
Histone over-expression analysis. A: Effect of histone over-expression in hif1Δ, *hat1*Δ, *hat2*Δ and *lsm1*Δ mutant strains. Note: Growth on galactose turns *GAL1* promoter ON resulting in histone over-expression (O/E) B: Tetrad analysis of yeast strains. Yeast haploid strains *lsm1*Δ and *hif1*Δ, *hat2*Δ, and *hat1*Δ were crossed. Representative tetrads that were dissected for each cross are shown. Double mutants are shown as circles.

We found that *hif1*Δ and *hat2*Δ cells were mildly sensitive to H3 over-expression whereas *hat1*Δ cells were not affected (Figure 5A), suggesting a role of Hif1 and Hat2 proteins in histone homeostasis. We also observed that *hif1*Δ, *hat1*Δ, and *hat2*Δ cells were mildly sensitive to genotoxic agent hydroxyurea (HU) which stalls replication forks and can induce DNA DSBs after prolonged exposure (Petermann *et al.* 2010) (Figure S5). In accordance with a previous report (Herrero and Moreno 2011), cells lacking *LSM1* exhibited a hypersensitivity to histone H3 over-expression (Figure 5A) as well as to HU (Figure S5). Since *hif1*Δ, *hat2*Δ and *lsm1*Δ cells exhibit some degree of phenotype in response to H3 over-expression, we reasoned that Hif1, Hat2 and Lsm1 might be functionally related. To answer this question, we carried out tetrad analysis of crosses between haploid *lsm1*Δ and *hif1*Δ, *hat2*Δ, and *hat1*Δ cells. For each cross we dissected nine tetrads after sporulation. In contrast to *lsm1*Δ*hat1*Δ cells which grew normally, a severe phenotype of slow growth was observed for *lsm1*Δ*hat2*Δ and *lsm1*Δ*hif1*Δ segregants (Figure 5B). These observations suggest a synergistic functional link between Lsm1 and Hif1/Hat2 proteins.

**Figure 6:**
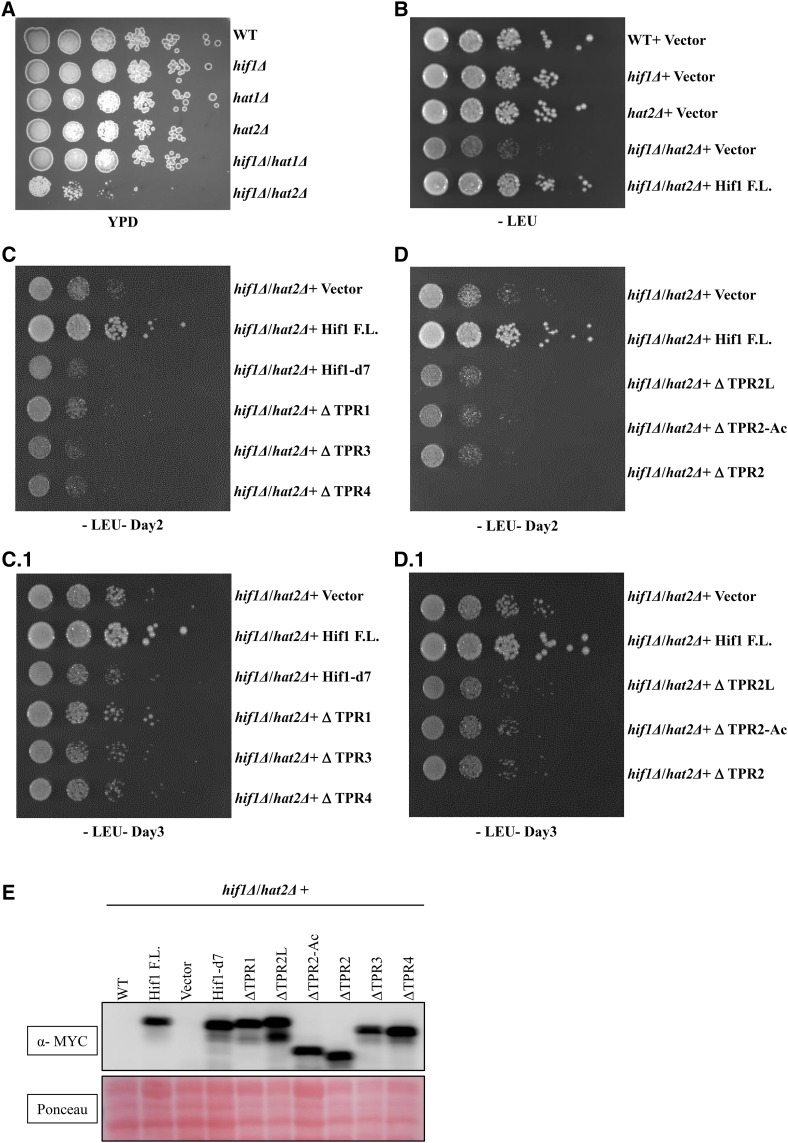
Analysis of hif1Δ hat2Δ double knockout cells for growth defects on YPD media. A: Strains were grown to an OD at 600nm of ≅0.5 before being plated at fivefold serial dilutions on YPD. B: Strains were cloned into pRB4151-2MYC lacking the Leucine amino acid (-Leu) for selectivity. Hif1 F.L. was transformed into hif1Δ/hat2Δ and hif1Δ to rescue the phenotype. C, D: Various Hif1 truncations were expressed back into hif1Δ hat2Δ double knockout cells to examine their ability to rescue the phenotype. Note: C1 and D1 represent 3 days of growth. E: Western blot analysis of whole cell extracts to examine the expression of Hif1 truncation mutants in *hif1Δ hat2Δ* double knockout cells.

To further establish the functional link between Hif1 and Hat2 proteins, we generated a double knockout lacking both the *HIF1* and *HAT2*. Interestingly, *hif1*Δ *hat2*Δ double knockout cells were synthetic sick and exhibited severe growth defects on YPD media (Figure 6). We then expressed back the above described truncation mutants into *hif1*Δ *hat2*Δ cells to examine whether they could rescue the observed phenotype. Interestingly, the full length Hif1 successfully rescued the phenotype, however, none of the examined truncation mutants were able to overcome the growth defects of *hif1*Δ *hat2*Δ cells (Figure 6). These results indicate the requirement of various Hif1 functional domains for its proper functioning. Taken together, the overall pattern of sensitivity to H3 over-expression and genetic interaction of Hif1/Hat2 with Lsm1 suggest that these proteins presumably function synergistically to maintain appropriate histone levels.

### Hif1 is critical for chromatin-related processes in multiple contexts

To gauge the functions of Hif1 in a broader context we constructed its interaction network using the available protein-protein interaction and genetic interaction data (Figure S7A) (Krogan *et al.* 2006). The network indicated that in addition to histones H3/H4, Hat1/Hat2 and Asf1, Hif1 has potential functional links with several genes/proteins involved in a wide range of chromatin-related processes. Notably, we found that Hif1 has links with 1) regulators of gene expression such as TAS1, SIR5, SAS2, SAS4, and Rtt106; 2) chromatin assembly proteins including CAF-1, HIR-complex and chromatin remodelers such as FACT; 3) DNA replication associated proteins including ORC, RFC, Cdc45and MCM2; 4) DNA damage response protein Rad53 which has a defined role in excess histone degradation in a proteasome-dependent manner; 5) H2A/H4 histone acetyletransferease complex; 5) transcription regulation related proteins such as Spt2 and Bdf1; 6) RNA polymerase II transcriptional pre-initiation complex assembly and TAF2, TAF7 and TAF13. Among Hif1 physical interactions Asf1 and Hat1 have been extensively reported (for review 17), and thus we included their sub-networks in our analysis as well (Figure S7B). The widespread functional links of Hif1 are akin to those of Hat1/Hat2 and Asf1—a key generalized H3/H4 chaperone, suggesting that Hif1 is a critical component of various chromatin assembly and disassembly processes (Figure S7C).

**Figure 7:**
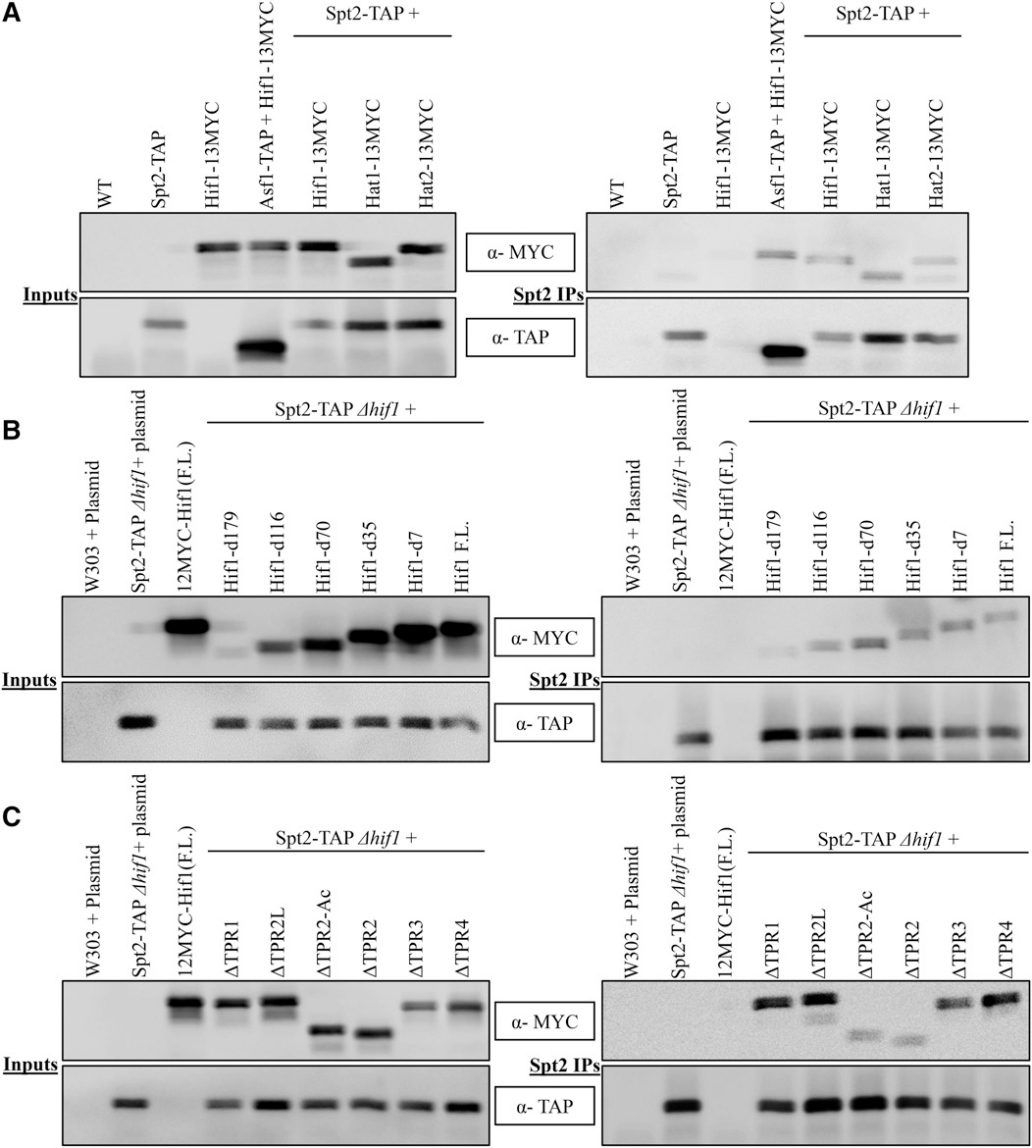
Western blot analysis of Co-IP fractions of Spt2 for its ability to immuno-precipitate with three subunits of the Hat1-complex. A: *(Left)* Input fractions of Co-IP experiments. *(Right)* Co-IP samples of Spt2-TAP. Asf1-TAP was used as a control in Co-IP experiments to capture Hif1-Asf1 interaction B: *(Left)* Input fractions of Co-IP experiments for various Hif1 C-terminal (external) deletions. *(Right)* Western blot analysis of Co-IP fractions of Hif1 C-terminal mutants to assess their ability to immunoprecipitate with Spt2-TAP. C: *(Left)* Input fractions of Co-IP experiments for various Hif1 internal deletion mutants. *(Right)* Co-IP samples of Hif1 internal deletions.

While a detailed analysis of Hif1 functional interactions awaits future studies, Spt2 which is present as a shared node between Asf1 and Hif1 (Figure S9B) drew our attention due to its critical role as a negative regulator of transcription initiation (Peterson *et al.* 1991; Bhat *et al.* 2013). Spt2 has been linked with nucleosome re-assembly during transcription (Thebault *et al.* 2011). It travels along with RNAP II and functions in transcription elongation (Nourani *et al.* 2006). Its presence in the Hif1 network prompted us to investigate the functional aspects of this interaction. We first established the physical interaction between Spt2 and the members of Hat1-complex. These interactions have previously been reported in large scale protein-protein interaction studies (Krogan *et al.* 2006). To this end, *S. cerevisiae* cell lines with a genetic background of C-terminally TAP tagged Spt2 were engineered expressing either *HIF1*, *HAT1* or *HAT2* carrying C-terminal 13MYC epitope tag from their native chromosomal loci. Subsequently, using Co-IP strategy we analyzed the interaction between Spt2 and subunits of the Hat1-complex. Consistent with previous studies (Krogan *et al.* 2006) Hif1 as well as Hat1 and Hat2 proteins co-immunoprecipitated with Spt2 (Figure 7A) suggesting a stable interaction between Spt2 and HAT1-complex.

To identify the region of Hif1 required for an interaction with Spt2 *in vivo*, we employed our above described Co-IP strategy. Strikingly, similar to our above noted observations for Hat1/2, H3/4 and Asf1, we found the acidic region to be essential for Hif1 interaction with Spt2 (Figure 7B,C). These data, taken together, demonstrate centrality of the acidic region for proper Hif1 functioning.

## Discussion

NASP-family proteins have been implicated in a wide range of processes including buffering of soluble H3-H4 reservoirs (Cook *et al.* 2011), mediating H4 acetylation in the context of Hat1-complex (Poveda *et al.* 2004; Ai and Parthun 2004; Campos *et al.* 2010), and CENPA chaperone activities (Dunleavy *et al.* 2007). In humans NASP expression has been reported to be significantly altered in many cancers including prostrate (Alekseev *et al.* 2011). In spite of these demonstrated roles of NASP-family proteins in various chromatin related processes (for review Finn *et al.* 2012), underlying mechanistic details are unclear. Here we have demonstrated that the acidic region present within the TPR2 of NASP homolog Hif1 in *S. cerevisiae* is critical for binding with the Hat1/Hat2-complex, Asf1 as well as histones H3/H4. We have also provided evidence for the possible involvement of Hif1 in the regulation of histone dynamics emphasizing the central role of NASP-family proteins in the general maintenance of chromatin.

### Conservation of Hif1 among fungi

We have examined Hif1 homologs with a conserved TPR motif architecture within all major fungal groups. This finding is consistent with our previous report (Nabeel-Shah *et al.* 2014) which established that NASP-family proteins are conserved throughout the major eukaryotic super-groups. NASP-family proteins share a common feature of being histone chaperones however a degree of functional diversity does exist across different species. A clear example of this functional diversity has been noted for closely related *S. cerevisiae* and *S. pombe* fungal lineages. *S. cerevisiae* Hif1 is a H3/H4 chaperone that binds with Hat1/Hat2 to mediate H4 acetylation (Parthun 2012) whereas *S. pombe* Sim3 is a CenpA specific chaperone and has been reported to functionally overlap with Asf1 (Dunleavy *et al.* 2007; Tanae *et al.* 2012). Consistent with these previous reports, our MSA analysis revealed lineage specific differences at key amino acid positions within various Hif1 homologs. For example, variable length insertions into TPR2 and TPR4 motifs argue in favor of functional diversity within closely related lineages.

The presence of large number of acidic residues interrupting TPR2 results in an overall net negative charge which appears necessary for an interaction with positively charged histones (Liu *et al.* 2014). The acidic region is critical for proper Hif1 function (Liu *et al.* 2014) and has been reported to be a subject of strong purifying selection (Nabeel-Shah *et al.* 2014). Several studies have experimentally established the functional importance of this TPR2 acidic interruption region. Considering its conservation during evolution, and taking earlier findings into account we suggest that the observed variations within the acidic interruption region likely accounts for the functional diversity previously reported within closely related fungal lineages (see above).

### Structural and functional aspects of Hif1

Hif1 has been extensively reported to function as a component of NuB4 complex which acetylates histone H4 (Parthun 2007, reviewed in 2012). Human sNASP also forms a complex with Hat1/Hat2 in the cytoplasm to mediate deposition-related acetylation of newly synthesized histones H4 (Campos *et al.* 2010). Despite the well conserved nature of Hif1-Hat1/Hat2 interaction across species, until now there had been a lack of information regarding the mechanistic details. In this study we have shown that the acidic region interrupting the TPR2 of Hif1 is essential for an interaction with Hat1 and Hat2. We cannot exclude the possibility that Hif1 mutant lacking the acidic region might bind to unmodified H4, however. our data indicates that acidic patch is absolutely required for an interaction with acetylated histones. Crystal structure of Hif1 showed that the TPR2 acidic region is crucial for H3/H4 binding (Liu *et al.* 2014). The overall structure of Hif1 forms a groove with acidic region surrounding the outer surface of the TPR (Liu *et al.* 2014). Previous evidence also indicates that Hif1 binds with H3/H4 and then recruits the Hat1/Hat2 complex (Campos *et al.* 2010). These observations combined with our results suggest a model for Hif1 function where H3/H4 held onto the outer surface of the acidic region are presented to the Hat1/Hat2 complex for HAT enzymatic activity and are then passed onto Asf1 for nuclear import.

In contrast to Hif1, the acidic region of human sNASP has been reported to be essential for ^1^H binding and TPR4 appears critical for H3/H4 interaction (Wang *et al.* 2012). Two possible reasons could account for this apparent discrepancy between sNASP and Hif1. First, *in vitro* data have suggested that the deletion of TPR4 in sNASP does not completely abolish H3/H4 binding affinity although it is significantly decreased (Wang *et al.* 2008; Bowman *et al.* 2016). This raises the possibility that human sNASP TPR2 acidic region may also participate in H3/H4 binding however ^1^H might be its preferred substrate. Second, molecular evolutionary analysis has indicated that TPR1 and TPR4 of NASP-family proteins have evolved more rapidly in comparison to the TPRs2/3 (Nabeel-Shah *et al.* 2014). In the light of these studies, it appears that over the course of evolution, mammalian NASP might have assumed ^1^H chaperone activity via acidic region whereas H3/H4 binding activity shifted to the TPR4 through adaptive evolution. This model of domain-specific functional evolution accommodates the observed nearly abolished yet not fully eradicated binding of H3/H4 in the TPR4 deleted sNASP (Wang *et al.* 2012).

The physical interaction of Asf1 and NASP-family proteins has been conserved across diverse eukaryotes including *Tetrahymena* (Garg *et al.* 2013). Asf1 is thought to function downstream of Hif1 in the H3/H4 transport pathway (Campos *et al.* 2010). *In vitro* studies have indicated that Hif1 can bind directly to the Hat1/Hat2-complex, independently of H3/H4. However Asf1 interaction is likely mediated by H3/H4 (Haigney *et al.* 2015). In this report, we have shown that the Hif1 acidic-interruption region of TPR2 is essential for interactions with both the H3/H4 as well as Asf1. This suggests that histones H3/H4 might in fact be mediating the *in vivo* Hif1-Asf1 interaction, consistent with previous *in vitro* reports (Haigney *et al.* 2015). While we cannot exclude the possibility of additional sites of interactions, our results indicate that the TPR2 acidic-interruption region is absolutely required for Hif1 proper functioning in the reported H3/H4 transport pathway (Campos *et al.* 2010). These observations also provide experimental support to our previously reported evolutionary analysis of NASP-family proteins suggesting that the acidic region interrupting the TPR2 have experienced an unusually strong form of purifying selection and thus might have significant impact on proteins’ overall functioning (Nabeel-Shah *et al.* 2014).

Hif1 was originally identified as a nuclear specific component of the Hat1-complex (Poveda *et al.* 2004). Human NASP has also been found primarily as a nuclear protein (Richardson *et al.* 2000; Alekseev *et al.* 2003). Accordingly, some studies have predicted the presence of a C-terminal NLS among NASP-family proteins (Finn *et al.* 2012). We have systematically aligned Hif1 C-terminal basic patch with the consensus NLS sequence and experimentally established its functionality. To our knowledge this is the first report to demonstrate the presence of a functional NLS in the NASP family proteins among fungi. Interestingly, we have recently shown that a basic patch found at the C-terminus of a fungal specific HAT Rtt109 is required for *in vivo* H3K9 acetylation activity (Radovani *et al.* 2013). In contrast to Rtt109 however, the C-terminal basic patch (or NLS) of Hif1 does not appear to be required for Hat1/Hat2 binding activity. It is currently unknown whether the C-terminal basic batch of Rtt109 functions as an NLS. Similarly, in humans the C-terminus of sNASP is not required for binding with the histones and for *in vitro* nucleosome formation activity (Osakabe *et al.* 2010; Wang *et al.* 2012). Several recent studies have suggested that in addition to its nuclear roles Hif1 also interacts with the cytosolic Hat1/Hat2 complex (Blackwell *et al.* 2007; Campos *et al.* 2010). The fact that a C-terminally truncated Hif1 mutant is able to immunoprecipitate with Hat1, Hat2 and H3/H4 provides support for the presence of a functional cytosolic Hat1-Hat2-Hif1 complex. Interestingly, it has previously been proposed that Hif1 passively enters the nucleus in the context of a multi-protein complex (Hat1-Hat2-Hif1-H3/H4-Asf1) which recruits KAP123 (17). This model implies that deleting Hif1 NLS would not abolish its nuclear localization. In contrast, however, our observation that deleting Hif1 NLS does in fact abolish its nuclear localization suggests for the existence of a separate Hif1 nuclear entry pathway. Furthermore, Hif1-truncated mutants (ΔAcd /ΔTPR2) defective for an interaction with the Hat1/Hat2 are fully functional for their proper nuclear import suggesting that Hif1 nuclear import is independent of the Hat1-Hat2 complex.

An excess of histones can be highly toxic to cells (Singh *et al.* 2010), and hence histone gene expression is tightly regulated throughout the cell cycle (Kurat *et al.* 2011, 2014). NASP-family proteins including *X. laevis* N1/N2 and human NASP have been demonstrated to function as a buffer for the maintenance of soluble histones H3/H4 proteins (Dilworth *et al.* 1987; Cook *et al.* 2011). In this study we show possible similar functions of Hif1 in the regulation of histones. Our genetic interaction data highlights a synergistic functional link between *HIF1* and *LSM1* which has previously been shown to regulate histone mRNAs levels (Herrero and Moreno 2011). Our unpublished chromatin immmuno-precipitation (ChIP) data indicate that Hif1 does not localize to histone *HTA1-HTB1*genomic regions. Further, mRNA levels of *HTA1* were not significantly affected in a *hif1Δ* mutant thus excluding the possibility of a direct role in regulating histone gene expression (J. Fillingham *et al.* unpublished results). We suggest that similar to human NASP, Hif1 functions in buffering the soluble histones H3/H4 proteins. Our data supports a potential role of Hat2 protein in this pathway. Additional studies will be required to fully underscore the exact nature of Hif1 and Hat2 roles in regulating histone metabolism. To this end, studying the role of Hif1 in the context of Rad53 which is critical for excess histone degradation (Gunjan and Verreault 2003) and has been detected in the Hif1 interaction network will be useful. Furthermore, examining the role of various TPR motifs that appear to be dispensable for histone binding will be informative. The intriguing fact that none of the deletion truncations were able to rescue *hif1Δ hat2Δ* double mutant phenotype is suggestive of functional importance of various TPRs. In particular, deciphering the functional aspects of TPR4 and the insertion that it harbors will be useful to provide a more comprehensive view of Hif1 roles. In fact, TPR1 and TPR4 have evolved more rapidly than TPRs2/3 (Nabeel-Shah *et al.* 2014) suggesting that these motifs might be subject of adaptive evolution resulting in the acquisition of new functions.

We have assessed the possible involvement of Hif1 in a wide range of chromatin-related processes. Of note, human NASP has been implicated in a variety of processes including histone transport (Campos *et al.* 2010), DNA replication (Alekseev *et al.* 2003, 2005; Richardson *et al.* 2006) as well as stem cell proliferation (Yocum *et al.* 2008). Our gene/protein functional network data implicate Hif1 in an array of cellular processes. To this end, we have provided the initial evidence for the role of Hif1 in gene transcription. Hif1 physical interaction with a transcription regulatory protein Spt2 suggests that Hif1 might be important for chromatin reassembly during transcription. Spt2 has been shown to prefer a chromatin assembly pathway that uses old histones removed by RNAP II (Thebault *et al.* 2011). We propose that Hif1 buffers displaced histones during transcription and makes them available for immediate chromatin reassembly. This model is consistent with the known roles of Hif1 with respect to the nucleosomal assembly (Ai and Parthun 2004), H3/H4 binding activities, and previously established role of components of NuB4 complex in histone turnover (Verzijlbergen *et al.* 2011). Further analysis will be required to understand the mechanistic details of this model. For example, determining how Hif1 and Spt2 co-operate to regulate transcription and what exactly is the role of Hat1/Hat2 subunits of the NuB4 in this process will be informative.
